# Maternal sepsis complicating arabin cervical pessary placement for the prevention of preterm birth: a case report

**DOI:** 10.1186/s12884-016-1209-0

**Published:** 2017-01-17

**Authors:** Begoña Martinez de Tejada

**Affiliations:** Obstetrics Unit, Department of Obstetrics and Gynecology, Geneva University Hospitals and Faculty of Medicine, 30 Boulevard de la Cluse, 1205 Geneva, Switzerland

**Keywords:** Maternal sepsis, Cervical pessary, Medical complications, Prevention, Preterm birth

## Abstract

**Background:**

Preterm delivery is a major health problem and contributes to more than 50% of all neonatal and infant deaths. Recently, there has been a renewed interest in the use of cervical pessaries as a safe and effective intervention with few maternal side-effects for the prevention of preterm birth in both single and twin pregnancies.

**Case presentation:**

A 43-year-old gravida 5, para 1 (previous preterm birth at 24 weeks) patient with an in vitro fertilization twin pregnancy had an Arabin cervical pessary placed at 19 weeks of pregnancy due to the presence of cervical funneling identified by ultrasound screening. She developed chorioamnionitis and sepsis and delivered at 21 3/7 weeks after extraction of the pessary.

**Conclusion:**

Severe maternal infection may complicate pessary treatment for the prevention of preterm birth. Careful follow-up is necessary in women with a cervical pessary during pregnancy, particularly when important funneling is present.

## Background

Preterm birth (PTB) is defined as delivery before 37 completed weeks of gestation and is a major contributor to perinatal mortality. Among all causes of perinatal mortality, 50–70% can be attributed to PTB. Similarly, PTB is the leading cause of neonatal morbidity, mostly due to respiratory immaturity, intracranial hemorrhage, and infections. These conditions can result in long-term neurodevelopmental sequelae, such as intellectual impairment, cerebral palsy, chronic lung disease, deafness, and blindness [1, 2]. Thus, prevention of spontaneous PTB remains one of the biggest challenges in obstetric care.

Vaginal pessaries have been used for centuries to treat uterine or vaginal vault prolapse. Their use for the prevention of spontaneous PTB has been mostly quite occasional until the recent publication of a randomized clinical trial that showed a reduction in the rate of PTB before 34 weeks in the group of women with a short cervix treated with cervical pessary compared to the non-treated group [3]. Cervical pessary has been shown also to reduce the rate of poor neonatal outcomes in women with twin pregnancies and a cervical length of less than the 25^th^ percentile (38 mm) [4]. Pessaries are described as safe to be used in pregnancy with a chorioamnionitis rate of around 3%. We present here a case of severe chorioamnionitis and maternal sepsis complicating cervical pessary treatment for the prevention of PTB in a patient with a twin pregnancy [3, 4].

## Case presentation

**Fig. 1:**
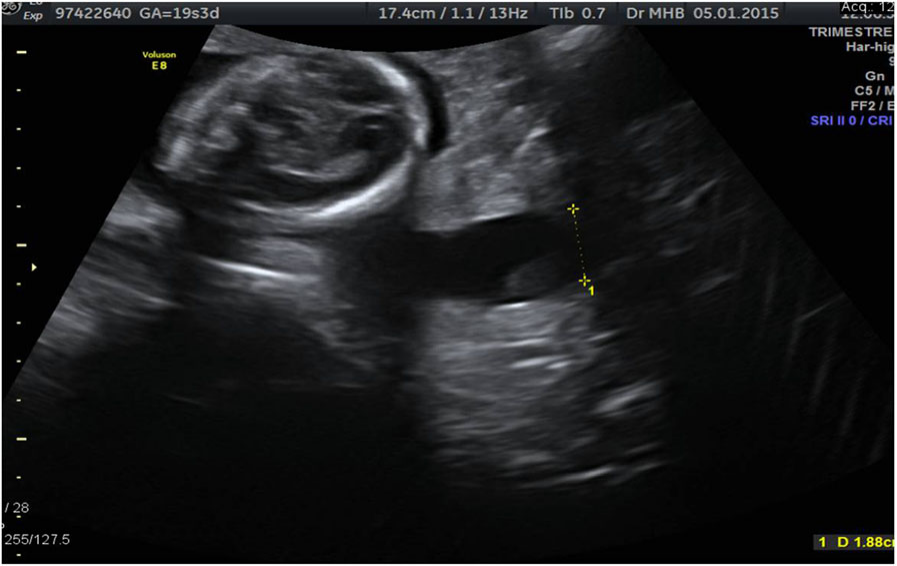
Cervical ultrasound at 19 3/7 weeks showing a long and open cervix

A 43-year-old gravida 5, para 1 (previous preterm birth at 24 weeks) patient with a twin pregnancy from in vitro fertilization (IVF). Her past medical history was significant for three previous first trimester miscarriages (the latter with a cervical cerclage) and a preterm delivery at 25 weeks’ gestation of a girl of 1000 g who died at 1 month of life in Madagascar. Due to an infertility problem for the past 3 years, the patient had undergone IVF in Spain to get pregnant. An ultrasound performed at 7 weeks confirmed a dichorial-diamniotic twin pregnancy with a long cervical length (>40 mm). She had been placed on hormonal treatment (estrogens and progesterone) during the first 14 weeks of pregnancy. The cervix had been checked again at 9, 12, and 16 weeks both clinically (long, posterior, and closed) and sonographically (>40 mm and without funneling). However, during a regular pregnancy visit at 19 3/7 weeks, the ultrasound examination showed a long cervix (48 mm), but open at about 18 mm along all of its length (Fig. 1). Clinically, the patient was asymptomatic (no uterine contractions, no signs/symptoms of infection) and her uterine cervix was long and almost closed (the membranes were not visualized through the external cervical os [ECO]). We decided to place an Arabin pessary after performing wet mount and vaginal and cervical cultures (all examinations were negative). One week later (20 3/7 weeks), she came back for a check-up. The pessary was well tolerated and well positioned at speculum examination; the ECO was 1 cm open and the fetal membranes were visualized through it, but not bulking.

**Fig. 2:**
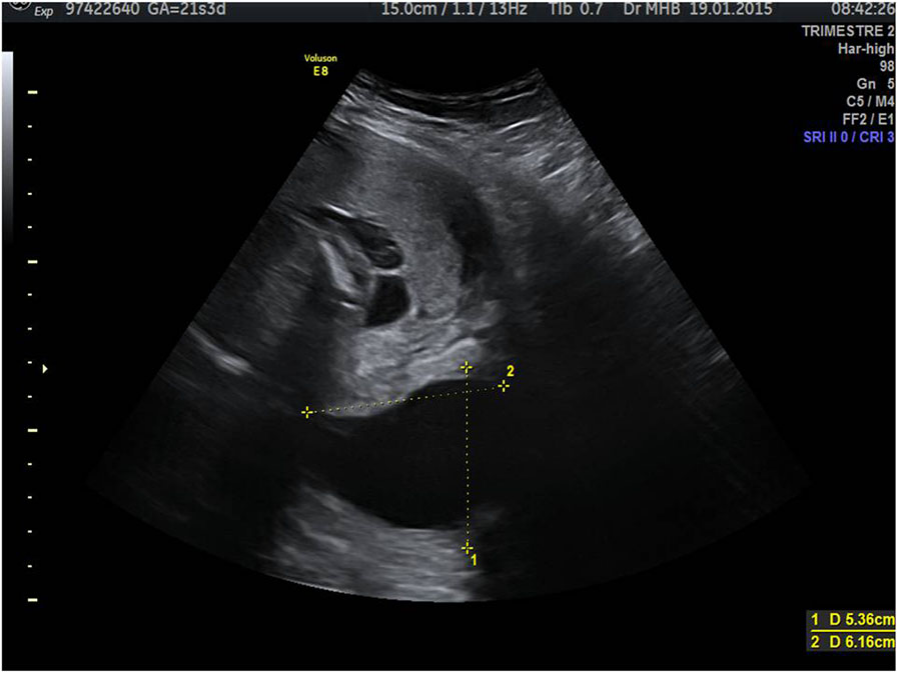
Cervical ultrasound at 21 3/7 weeks showing a long cervix with bulging membranes through the pessary

At 21 2/7 weeks, the patient consulted the emergency unit for suspicion of rupture of the membranes. At clinical examination, rupture of the membranes was excluded. The pessary was well positioned, but the membranes were bulking through the ECO and around 2 cm in the vagina. The ultrasound showed normal amniotic fluid, normal growth of both fetuses, and a long (58 mm), but open cervix (approximately 4 cm) (Fig. 2). The patient had no uterine contractions, no fever, vaginal secretions were normal and the blood examinations showed no leucocytosis (12 G/l and 6.7 G/L 6 h later), but high C-reactive protein (CRP) levels (142 mg/l and 114 mg/l 6 h later). The patient was hospitalized for a threatened second trimester miscarriage. Several hours later, the patient started to shiver and developed a high temperature (39.5 °C). She complained about slight abdominal pain and rupture of the membranes occurred. A blood test showed high leucocytes (18.5 G/l, 9.5% non-segmented) and a high CRP (242 mg/l). The patient was transferred to the labour suite, put on intravenous antibiotics (amoxicillin and gentamicin) and the pessary was removed. The cervix was 1 cm long and 1 cm dilated. Two hours later, the cervix was dilated at about 4–5 cm.

The patient delivered the first baby, a stillborn girl (395 g) in breech presentation, followed by delivery of the second twin, a live boy of 480 g, after artificial rupture of the membranes. The baby died afterwards. The patient developed septic shock, with hypotension and bradycardia. She was transferred to the operating theatre to remove the placentas after intubation and then to the intensive care unit where she continued the antibiotic treatment (clindamycin had been added before placental removal). She was transfused and extubated 18 h later. She was put on continuous positive airway pressure and respiratory physiotherapy due to lung oedema. Blood cultures became negative and placenta culture became positive for vaginal flora. She was transferred to the maternity unit 24 h later where she continued the respiratory physiotherapy and the antibiotic treatment was switched to amoxicillin/clavulanic acid orally after 48 h without fever. The histological examination of the placenta showed acute and diffuse choriomnionitis and vasculitis.

## Discussion

PTB occurs in approximately 7–8% of all live births in Switzerland [5]. This rate is much higher in some developed (12% in the USA) and developing countries (18%). PTB is the major determinant of adverse neonatal outcome and infant mortality rates range from 400 per 1000 live births at 24 weeks’ gestation to 1.4 at term [2]. Long-term sequelae in PTB survivors include motor, visual, auditory and cognitive impairment, epilepsy, behavioural problems, and increased risk of hospital readmissions [6, 7]. PTB and its consequences can have a negative emotional and psychosocial impact on parents and families, which can last well beyond the initial period of hospitalisation of the preterm baby [1]. The financial consequences for the public sector of caring for children born preterm are also a significant burden. [8]. Twins, with a prevalence of 1.5% of pregnancies, account for approximately 25% of all PTBs.

Recently, the application of a cervical pessary for the prevention of PTB has generated renewed interest. The Arabin pessary is the most frequently used for this indication. The pessary is supposed to function not only by preventing further opening of the internal os, but also by changing the inclination of the cervical canal relative to the uterus. In turn, this may prevent direct pressure on the membranes as the weight of the uterus is directed more towards the lower anterior uterine segment. It has been shown that pessary placement increased cervical oedema, which could prevent cervical effacement [9]. Of note, it is recommended not to remove the pessary after premature rupture of the membranes, which is known to be a risk factor for chorioamnionitis.

A randomised controlled trial to investigate whether cervical pessary use could prevent PTB was published in the *Lancet* in 2012 [3]. The study included 385 women with singleton pregnancies and a cervical length less than 25 mm and showed a significant reduction in the rate of PTB at less than 34 weeks (6 vs 27%; odds ratio, 0.18 [95% CI 08–0.37]; *p* <0.001) in the pessary group. The authors showed also a significant reduction in the rate of composite neonatal adverse events (3 vs 16%; odds ratio, 014; [95% CI 0.04–0.39]; *p* <0001). The rate of maternal side-effects was similar between the pessary and the non-pessary groups, except for the rate of vaginal discharge, which was 100% in the pessary group. The rate of choriomnionitis was 3% in both groups. No maternal sepsis was reported. In 2013, another randomised controlled trial was also published on the same topic in the *Lancet*, but this time including twin pregnancies [4]. In this study, 813 women were randomised to pessary or no treatment. There was no difference in the rate of PTB at less than 37, 32 or 28 weeks or in the rate of adverse neonatal outcomes. In a secondary analysis including women with a cervical length less than the 25^th^ percentile (38 mm), poor perinatal outcome was less frequent in the pessary group (10%) than in the control group (24%; *p* = 0.01). Median gestational age at delivery was also longer in the pessary group than in the control group (36.4 vs 35 weeks, respectively; *p* = 0.04). The rate of chorioamnionitis was 3% in both groups and there was one case of maternal death in the pessary group (no reason reported).

We took the decision to insert the pessary in our patient as the risk for late miscarriage or PTB was high based on the progressive opening of the cervix. We did not perform a cerclage as current guidelines do not recommend cerclage in twin pregnancies as a higher risk of chorioamnionitis and PTB is reported when the procedure is performed in this setting. When the pessary was placed, our patient was asymptomatic and the clinical cervical examination was normal (slight opening of the ECO); membranes were not bulging. Interestingly, other authors have reported the beneficial effect of the pessary even when placed in women with bulging membranes [9, 10]. PTB is often associated with intrauterine infection and this risk is higher when decreasing the gestational age at delivery [11]. It is unknown whether our patient had intrauterine infection leading to PTB as we did not perform amniocentesis. We postulate that the pessary prevented the cervical canal from further effacement and dilatation and thus avoided spontaneous miscarriage. As the membranes bulged through the pessary and were exposed to the vaginal flora, the patient developed chorioamnionitis and sepsis. If the pessary had not been placed, our patient would probably have expulsed the fetus earlier and maternal sepsis could have been prevented. Once the pessary was removed, cervical dilatation and fetal delivery happened very rapidly.

Cervical pessary may be an effective tool for the prevention of PTB as shown in several studies [3, 4, 9, 12]. Nevertheless, uncertainty about its efficacy persists as other studies have not shown any benefit from the placement of a cervical pessary in singleton or twin pregnancies [13, 14]. We recommend that pregnant women at high risk for maternal infection, such as those with important cervical funneling or bulging of the membranes, should be carefully followed for early diagnosis of infection and the pessary removed promptly following the appearance of any sign of infection.

## Conclusions

Clinicians should be aware of a possible risk of severe maternal infectious complications in pregnant women with placement of a cervical pessary. The pessary should be removed immediately in the case of a suspicion of infection in order to prevent severe complications.

## Abbreviations

CRP: C-reactive protein
ECO: External cervical os
IVF: In vitro fertilisation
PTB: Preterm birth
